# Parkinson’s disease beyond the brain: implications for treatments

**DOI:** 10.3389/fnagi.2025.1600782

**Published:** 2025-10-09

**Authors:** Setareh Malekian Naeini, Marc Danzell Lopez, Paul E. Fraser, Anurag Tandon

**Affiliations:** ^1^Department of Physiology, University of Toronto, Toronto, ON, Canada; ^2^Tanz Centre for Research in Neurodegenerative Diseases, University of Toronto, Toronto, ON, Canada; ^3^Department of Medicine and Medical Biophysics, Toronto, ON, Canada; ^4^Department of Medicine, University of Toronto, Toronto, ON, Canada

**Keywords:** synucleinopathy, enteric neurons, aggregation, lewy bodies, gastrointestinal tract

## Abstract

The presence of *α*-synuclein pathology in peripheral nervous system neurons is linked to early prodromal, non-motor symptoms in a segment of Parkinson’s disease (PD) patients referred to as “body-first.” These features of the disease suggest a convergence of environmental, genetic, immune and age-related factors within the enteric nervous system as initiating triggers of disease. This review explores the changes in the gut microbiome and intestinal permeability that may drive systemic inflammation and precede neurodegeneration in PD. The pathways leading to the formation of *α*-synuclein aggregates are explored as well as their role in transneuronal propagation and the spreading of pathology within the brain. Lastly, advances in systemic gene therapy that could be used to target multiple PD-affected organs following systemic injection are highlighted. By integrating insights from molecular biology and clinical research, it may be possible to shed light on the multifactorial etiology of PD and the interconnectedness of the gut and brain, which could lead to novel diagnostic and therapeutic strategies.

## 1 Introduction

Parkinson’s disease (PD) is a neurodegenerative disorder that is estimated to affect 12.0 million people by 2040 (Dorsey et al., 2018). The disease exerts a substantial economic burden; in the U. S. alone, the direct and indirect costs of PD summed up to $51.9 billion in 2017 (Yang et al., 2020). Therefore, understanding the underlying mechanisms of disease is essential in providing care to patients and reducing the economic burden on healthcare systems.

PD manifests clinically with motor disturbances including resting tremor, muscle rigidity, bradykinesia, and gait instability (Halliday et al., 2011). Some patients also experience non-motor symptoms such as rapid eye movement (REM) sleep behavior disorders (RBD), depression, and constipation prior to the appearance of motor deficits that elicit a PD diagnosis (Postuma and Berg, 2016). These prodromal symptoms implicate the involvement of multiple brain regions and the peripheral nervous system prior to substantia nigra degeneration. The triggers for PD remain unknown, though a combination of aging, genetics, and environmental factors are involved (Pang et al., 2019). These factors contribute to the toxic aggregation of proteins, most commonly the synaptic protein *α*-Synuclein (α-Syn), that disrupts neuronal homeostasis and leads to apoptosis. α-Syn aggregates found within the soma or along axodendritic projections are termed Lewy bodies (LB) and Lewy neurites (LN), respectively, and are associated with neuronal dysfunction. Thus, the principal pathological change in PD is the *α*-Syn aggregate-driven impairments and degeneration leading to the loss of dopaminergic neurons within the substantia nigra, a midbrain region involved in regulating movement via projections to the striatum (Halliday et al., 2011; Sonne et al., 2022).

The Braak gut-brain axis hypothesis of PD could explain the involvement of the peripheries, particularly the enteric nervous system (ENS), in the initiation and progression of disease (Beach et al., 2021; Braak et al., 2003). Entry of foreign pathogens through nasal or gut pathways could initiate or exacerbate pathology in enteric neuron and its passage via the vagus nerve to the brainstem (Braak et al., 2003; Braak et al., 2003). The presence of gastrointestinal (GI) symptoms such as constipation, dysphagia, gastroparesis, and excessive strain during defecation before the appearance of motor symptoms indicates early GI dysfunction and ENS involvement in PD (Sung et al., 2014; Gan et al., 2018; Yu et al., 2018). Lewy bodies (LBs) have been detected in the submucosal and myenteric layers of the GI system of PD patients mostly in the vasoactive intestinal peptide (VIP)-positive cholinergic neurons and minimally in tyrosine hydroxylase (TH)-positive neurons (Wakabayashi et al., 1988; Wakabayashi et al., 1989). Notably, one-third of age-matched non-parkinsonian patients also displayed enteric LBs and were suggested to have been in the premotor phase of PD (Wakabayashi et al., 1988; Wakabayashi et al., 1990). Enteric LBs have also been observed in the esophagus (Braak et al., 2006), as well as in nerve fibers of gastric, duodenal, and colonic biopsies of pre-clinical stage patients (Hilton et al., 2014).

It is important to note this staging for PD progression does not apply to all PD cases (Braak et al., 2003; Parkkinen et al., 2008). Conversely, enteric LBs are not detected in all PD patients nor do they serve as a sensitive or specific biomarker for PD (Chung et al., 2016; Ganguly et al., 2021; Visanji et al., 2015). A study examining 111 PD patients found minimal enteric LBs in only 5 patients. Since enteric LBs were absent in those without brain pathology, the researchers suggested that Parkinson’s disease may initiate solely in the brain (Beach et al., 2021). Such discrepancies between findings may be better accommodated by the hypothesis that postulates the existence of at least two subtypes of PD based on the origin of *α*-Syn pathology: body-first and brain-first. Patients of the body-first model are predicted to experience GI symptoms, cardiac denervation, and RBD prior to motor symptom onset while the opposite order is experienced by those of the brain-first phenotype (Borghammer and Van Den Berge, 2019; Borghammer, 2021). Although the numbers vary between studies, approximately 50% of premotor PD patients report constipation as compared to ~10% in age-matched controls with an even greater overrepresentation in the PD groups when broader GI symptoms were included (Cersosimo et al., 2013). Nevertheless, the periphery is still affected in both subtypes, whether in premotor or in the late stages of PD. This review aims to summarize how the disease may progress from the periphery to the brain, highlighting contributing factors such as gut dysbiosis and inflammation, and suggests the use of systemic AAV-mediated gene delivery as a potential therapeutic strategy that may concurrently treat the peripheral and central pathologies.

## 2 Gut dysbiosis

For patients of the body-first phenotype, disturbances to the gastrointestinal (GI) system are commonly observed in the premotor phase of PD. Constipation and inflammation are the most common gut symptoms affecting 50–60% of cases and about 25% of all PD cases develop these before motor symptoms (Gan et al., 2018; Yu et al., 2018). GI diseases involving inflammation like inflammatory bowel disease and Crohn’s disease also increase the risk of developing PD (Yu et al., 2018; Chen et al., 2019; Zhu et al., 2022; Fasano et al., 2015). A key player that may drive or counteract these symptoms is the gut microbiome.

The gut microbiome is composed of diverse microorganisms involved in metabolism, immunity, and regulation of the intestinal barrier (Tan et al., 2022). Microbial imbalance, termed dysbiosis, results in an alteration of microbial families present and the metabolites they produce, which can affect gastrointestinal and neurological processes (Tan et al., 2022). Thanks to technological advances such as next-generation sequencing, clinical studies have been able to identify the differences between the gut microbiota of PD and healthy patients using stool samples (Barichella et al., 2019; Cirstea et al., 2020; Tan et al., 2021). These studies have revealed deleterious taxonomic and metabolic shifts in PD patients that could be associated with constipation (Cirstea et al., 2020; Tan et al., 2021) and cognitive deficits. The importance of having a healthy microbiome has been highlighted by “gut reset” experiments wherein a healthy microbiome is introduced to subjects, commonly through a stool transplant (also known as fecal microbiota transplant). For example, in transgenic mice overexpressing *α*-Syn, transplantation of fecal microbiota from PD patients leads to worsened motor dysfunction and increased brain pathology compared to those given healthy fecal transplants, indicating the involvement of the microbiota in neurodegeneration (Sampson et al., 2016). Another study that employed a 1-methyl-4-phenyl-1,2,3,6-tetrahydropyridine (MPTP) induced mouse model of PD found that stool transplants from wild-type mice alleviated motor symptoms, improved gut dysbiosis, and increased the number of striatal dopaminergic neurons (Sun et al., 2018). Lastly, two case studies that tested the gut reset in PD patients also found beneficial effects on motor symptoms, bowel movements, mood, and sleep (Huang et al., 2019; Xue et al., 2020).

Although decoding the changes in the microbiome in PD is complicated, there is an overall increase in gram-negative bacteria that produce the endotoxin lipopolysaccharide (LPS). LPS damages the intestinal barrier, rendering it more permeable (more commonly known as a “leaky gut”) (Tan et al., 2022). Injection of LPS to mice has been shown to increase intestinal permeability and elevate the expression and pathological phosphorylation of *α*-Syn in colonic neurons, which are also observed in PD patients (Kelly et al., 2014). In another study, oral administration of LPS to mice over-expressing α-Syn led to the emergence of motor impairments (Gorecki et al., 2019). Gram-negative bacteria could also lead to LPS-mediated damage, as demonstrated by the administration of a gram-negative bacterium, *Proteus mirabilis*, which induced motor deficits, neuroinflammation, dopaminergic neuron loss, and α-Syn aggregation in the intestine and brains of mice (Choi et al., 2018). These pathological changes were attributed to LPS-mediated damage to the gut barrier and inflammation. Indeed, LPS mouse models of PD have emerged with a variety of administration routes, including nasal, oral, systemic injection, and localized injection in the substantia nigra and the striatum (Deng and Bobrovskaya, 2022). Impaired gut permeability, as demonstrated by mucosal α-Syn expression and serum LPS binding protein detected in PD patients, is indicative of the translocation of microbial byproducts (Bhattacharyya et al., 2019; Forsyth et al., 2011; Kim et al., 2016).

LPS could drive pathology through multiple mechanisms. Indeed, increased plasma LPS binding protein, a promoter of innate immunity and biomarker of intestinal permeability, has been associated with an increased risk for PD (Zhao et al., 2023). Endotoxins like LPS cause pathology through interactions with intestinal receptors, particularly toll-like receptor 4 (TLR4). TLRs are pattern-recognition receptors activated by endogenous damage or pathogens and triggering inflammatory responses implicated, for example, in inflammatory bowel disease (Tam et al., 2021). LPS interactions with intestinal TLR4s have been shown to mediate defects in epithelial tight junctions through TLR4’s downstream mechanisms, promoting gut leakiness and inflammation (Guo et al., 2015). Further, activation of TLR4 via LPS initiates a cascade of mechanisms that leads to pro-inflammatory gene expression and cytokine release, which further recruit leukocytes and enhance gut inflammation (Morris et al., 2015). This is supported by colonic samples from PD patients which revealed increased pro-inflammatory gene markers and cytokine expression (Perez-Pardo et al., 2019). In addition, TLR4 knock-out mice treated with a PD-inducing pesticide exhibited reduced intestinal inflammation, motor dysfunction, neuroinflammation, and neurodegeneration compared to wild-type mice, highlighting the importance of TLR4-mediated mechanisms in PD (Perez-Pardo et al., 2019). Studies have also shown that LPS can modulate *α*-Syn aggregation and initiate synucleinopathy *in vivo* (Kim et al., 2016).

Microbial alterations could also affect the metabolic byproducts produced by the microbiota, such as short-chain-fatty-acid (SCFA) levels. SCFAs, such as propionic acid and butyric acid, are produced when anaerobic bacteria digest fiber (Metzdorf and Tönges, 2021). SCFAs protect the intestinal barrier and reduce inflammation (Chen et al., 2018; Zhang et al., 2023). Some reports have shown an increase in some SCFA-producing genera and a simultaneous decrease in related microbiota in the fecal samples of PD patients as compared to healthy controls (Li et al., 2023). While the changes in bacterial strains may be due to PD drug interactions or natural ageing processes, these findings point to the complicated interactions of the microbiota and their role in human health. In addition, multiple studies have reported reduced SCFA levels in the stool samples of PD patients, while some studies show increased serum levels (Wu et al., 2022; Aho et al., 2021; Baert et al., 2021; Unger et al., 2016). Despite conflicting reports on SCFA levels, numerous *in vivo* studies have underscored the beneficial properties of SCFA in PD. Administration of sodium butyrate to a neurotoxin-induced mouse model of PD alleviated motor disability, inhibited neuroinflammation, and increased tyrosine hydroxylase levels in the substantia nigra (Hou et al., 2021). Similarly, intragastric administration of sodium butyrate into rotenone-treated mice protected against gut dysfunction and motor deficits (Zhang et al., 2022). Another study found that injection of *Blautia producta*, a butyrate-producing bacterium shown to be reduced in PD patients, improved motor deficits and attenuated dopaminergic neuron loss in a PD mouse model (Liu et al., 2024). Conversely, feeding SCFAs to germ-free mice over-expressing *α*-Syn exacerbated inflammation and motor dysfunction (Sampson et al., 2016). These contradictory findings may be attributed to the heterogeneity of PD and SCFAs, which require further elucidation via standardization of experimental protocols regarding SCFA type, SCFA dosage, and animal models used (Zhang et al., 2023).

The composition of gut microbiota can change through the process of ageing. Some of the effects of ageing on the gut include increased gut permeability, loss of microbial diversity, and a shift toward more harmful and inflammatory microbes (Cryan et al., 2019). For instance, housing young germ-free mice with older mice resulted in an increase in intestinal permeability and inflammatory markers in the gut of the germ-free mice due to microbial colonization with the microbiota from older mice (Thevaranjan et al., 2017). Germ-free mice were also shown to be resistant to neurotoxin-induced PD, however, they demonstrated motor dysfunction, gut permeability and intestinal inflammation after colonization by fecal bacterial content from older mice. These findings indicate a potential involvement of age-related gut microbiota dysbiosis in PD (Lima et al., 2023).

Beyond microbiota composition, certain bacterial proteins, such as the amyloid curli, can also be pathogenic. Curli fibers, composed of amyloid CsgA monomers, are expressed by commensal *E. coli* and are found in about 40% of human fecal isolates in the United States (Ravva et al., 2016; Miller et al., 2021). Curli is crucial for biofilm formation, which may occur after ingesting pathogenic microbial strains like *Salmonella typhi* and curli-positive *E. coli*. Notably, CsgA has a similar amyloidogenic structure to *α*-Syn fibrils and other related pathogens, enabling it to cross-seed the misfolding of α-Syn aggregates (Cherny et al., 2005; Evans et al., 2015; Lundmark et al., 2005). Indeed, aged Fischer 344 rats, *Caenorhabditis elegans*, and transgenics overexpressing α-Syn exhibited increased aggregation when fed a diet containing curli-positive bacteria (Sampson et al., 2016; Chen et al., 2016).

Overall, gut dysbiosis with increased LPS-producing bacteria contributes to gut permeability, allowing the translocation of microbial byproducts and gut contents into the underlying enteric neurons and into the systemic blood flow. This may promote the aggregation and spread of α-Syn as well as inflammation in the body-first PD cases.

## 3 Inflammation and PD

Gut inflammation has been demonstrated in PD patients with a pro-inflammatory microbial makeup and elevated stool levels of inflammatory markers, such as calprotectin, a marker of gut immune system activity (Klingberg et al., 2019; Mulak et al., 2019) and cytokines that include interleukin-1α, interleukin-1β, and C-reactive protein (Houser et al., 2018). Biopsies from the ascending colon of PD patients also reveal elevated mRNA expression levels of pro-inflammatory cytokines as well as the enteric glial cell markers, glial fibrillary acidic protein and Sox-10, consistent with enteric inflammation (Devos et al., 2013). This is in line with the finding that individuals with inflammatory bowel diseases have a higher risk of future PD development (Zhu et al., 2022; Li et al., 2023). In addition, anti-tumor necrosis factor therapy, a therapy for inflammatory bowel disease has been associated with a reduced risk of PD development through limiting peripheral inflammation (Peter et al., 2018). Therefore, an inflammatory gut environment is one of the homeostatic disturbances implicated in PD. As mentioned above, inflammation leads to increased gut permeability which could allow translocation of pro-inflammatory molecules, bacteria, bacterial metabolites, and amyloidogenic proteins to the underlying mucosal tissue where ENS neurons are located and subsequently into the circulatory system.

Systemic endotoxin translocation has also been reported in PD patients with lower levels of plasma LPS binding protein, an indicator of gram-negative bacteria exposure (Forsyth et al., 2011; Perez-Pardo et al., 2019). LPS can induce a systemic inflammatory response through the activation of monocytes and macrophages that produce pro-inflammatory cytokines (Kim et al., 2022). Indeed, studies on early PD patients have revealed the elevation of certain cytokines in the blood, like interleukin-2, interleukin-6, and interleukin-1ß in the early stages of the disease (Kim et al., 2018; Umemura et al., 2014). In fact, interleukin-2 and 6 were associated with non-motor progression, particularly mood/apathy-related symptoms (Kim et al., 2022). Systemic inflammation, whether through the leakage of deleterious gut contents or induced by gut inflammation, could in turn cause and exacerbate central neuroinflammation. For instance, infectious diseases like pneumonia are known to worsen the outcomes of PD patients and accelerate motor deterioration, due to systemic inflammation exacerbating neurodegeneration (Umemura et al., 2014; Brugger et al., 2015). In line with this, suppressing inflammation with nonsteroidal anti-inflammatory drugs has been shown to reduce the risk of future PD development (Chen et al., 2005; Chen et al., 2003).

**Figure 1:**
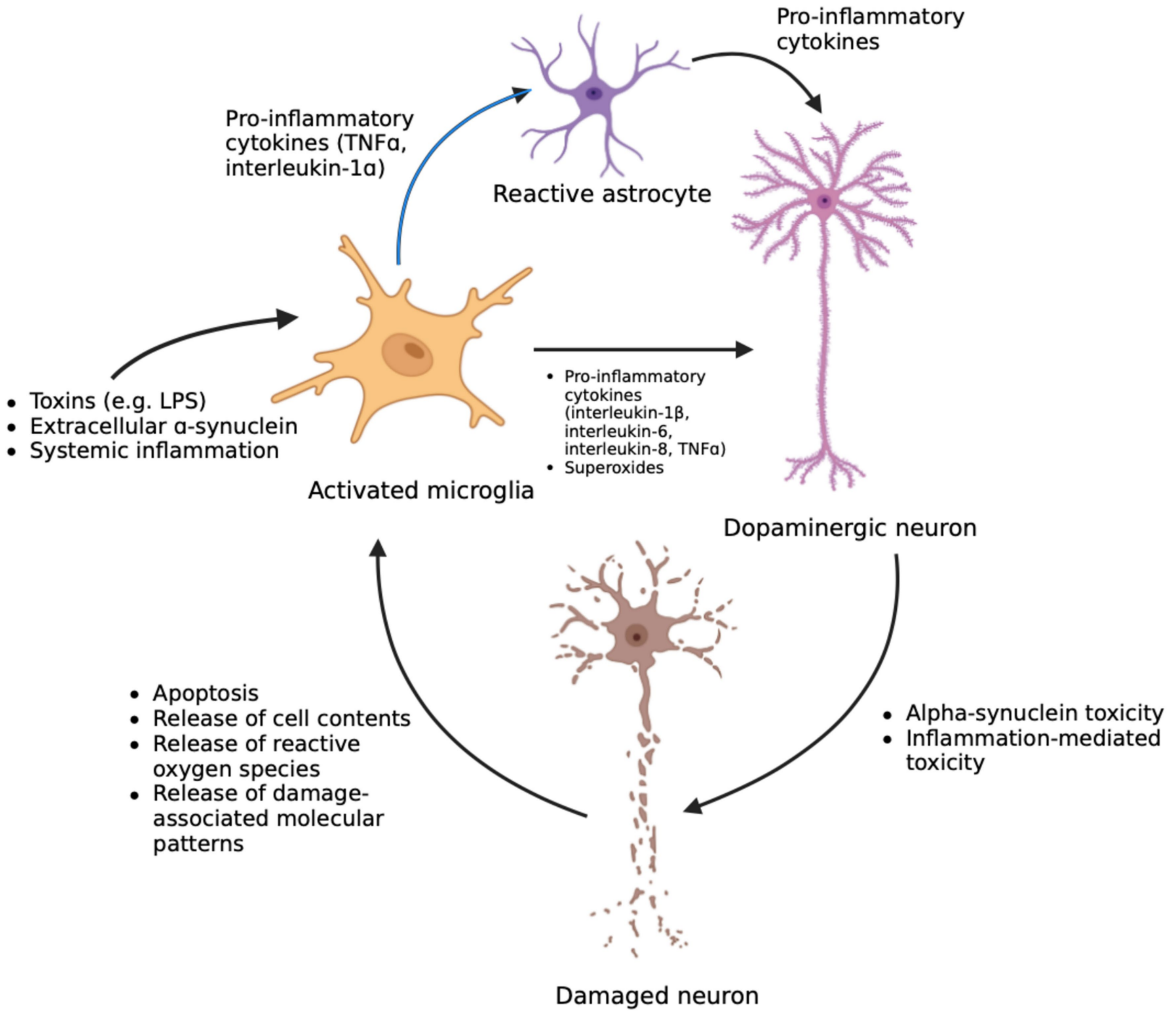
The cycle of neuroinflammation and neuronal damage in PD. Microglia are activated via circulating toxins, α-Synuclein aggregates released from cells, or systemic inflammatory agents crossing the BBB. Upon activation, microglia release pro-inflammatory markers and reactive oxygen species that activate astrocytes and mediate toxicity in neurons. Meanwhile, inflammatory toxicity further damages neurons that contain toxic alpha-Synuclein aggregates. This damage ultimately leads to cell death and the release of cell contents, reactive oxygen species, and more aggregates into the extracellular space which further activates microglia. This feed-forward cycle leads to exacerbated inflammatory responses and chronic neuroinflammation. Created in BioRender. Fraser, P. (2025) https://BioRender.com/tk5fbnp

Communication between immune pathways and the central nervous system (CNS) can happen in several ways (Figure 1). Firstly, inflammatory molecules must penetrate the protective blood–brain-barrier (BBB) – the selectively permeable membrane of the cerebral blood vessels – to enter the brain. While some cytokines like tumor necrosis factor-alpha (TNFɑ) and interleukin-1ß can cross the BBB through active transport, entry of leukocytes and other blood contents are restricted (Gao and Hong, 2008). Systemic inflammatory molecules could disrupt the BBB. For example, injection of rats with interleukin-6 and interleukin-2 significantly increased the blood-to-brain transfer constant, indicative of BBB permeability (Saija et al., 1995). Similarly, exposure to TNFɑ, interleukin-6, and interleukin-1ß in a rat cerebral endothelial cell model increased permeability, as determined by trans-endothelial electrical resistance (TEER), which measures the integrity of the membrane as an electrical barrier (de Vries et al., 1996). In addition, numerous studies have demonstrated the ability of endotoxins like LPS to disrupt BBB integrity. One study modelled infection by injecting *E. coli*, an LPS-producing bacterium, into rats and found an increase in BBB permeability through the vasodilatory mechanisms of nitric oxide (Shukla et al., 1995). Another demonstrated enhanced permeability of cultured cerebral endothelial cells treated with LPS, as indicated with reduced TEER (De Vries et al., 1996). A similar reduction of TEER was also observed in cultured rat brain endothelial cells treated with LPS, accompanied by increased oxidative stress and nitric oxide levels (Veszelka et al., 2007). Effects on BBB function have been reported in the substantia nigra of PD patients using magnetic resonance imaging, where a higher transfer rate of contrast agent into the brain compared to healthy controls indicated increased permeability attributed to microvasculature dysfunction (Al-Bachari et al., 2020). A meta-analysis also found BBB disruption in PD patients using biofluid markers, such as elevated ratios of albumin levels in cerebrospinal fluid (Wong et al., 2022).

A compromised BBB could increase the transfer of blood contents, including circulating toxins and systemic inflammatory molecules, into the brain parenchyma and induce neuroinflammation. Chronic neuroinflammation is implicated in neurodegeneration in PD and post-mortem analyses of substantia nigra and striatum revealed increased microglial activation, leukocytes, and the presence of inflammatory cytokines (Gao and Hong, 2008; Wong et al., 2022; Mogi et al., 1994; Brochard et al., 2009). Central inflammation and microglial activation were also observed in *de novo* PD patients compared to healthy controls (Yacoubian et al., 2023). Systemic inflammation and its effectors could induce neuroinflammation as shown by a mouse study where the systemic injection of TNFɑ or LPS caused microglial activation and drove cytokine production in the brain. Neuroinflammation was attributed to a systemic increase in TNFɑ which infiltrated the BBB through interactions with the TNFɑ receptor, without which neuroinflammation was not observed (Qin et al., 2007). Adaptive immunity could also infiltrate the brain, as evidenced by the presence of helper and cytotoxic T cells in the post-mortem substantia nigra of PD patients (Brochard et al., 2009). This investigation demonstrated the presence of fluorescent-tagged T cells in the substantia nigra and striatum of mice after neurotoxin-induced PD. This was accompanied by the leakage of a serum protein, albumin, in the brain which suggests that a leaky BBB contributes to the migration of leukocytes into the brain (Brochard et al., 2009). Systemic inflammation induced by gut dysfunction in an LPS-injected rat model of ulcerative colitis also exacerbated dopaminergic cell loss, microglial activation, serum and central cytokine levels, and BBB dysfunction and dopaminergic cell loss (Villarán et al., 2010). These data suggest that peripheral inflammation could migrate into the brain and modulate neuroinflammation, although, this is not a one-way interaction as central inflammation could also influence and recruit peripheral inflammation (Ferrari and Tarelli, 2011).

Another signal that promotes neuroinflammation comes from the contents of dying neurons. Apoptotic neurons release their cytoplasmic contents, including *α*-Syn aggregates, into the extracellular space which can signal microglial activation. This release results in phagocytosis and the activation of microglial downstream mechanisms that produce reactive oxygen species and further exacerbate dopaminergic neurotoxicity (Zhang et al., 2005). Dying cells also release damage-associated molecular patterns that are recognized by microglia (Calabresi et al., 2023). Microglial activation also promotes the release of pro-inflammatory cytokines, like interleukin-1β, interleukin-6, interleukin-8, and TNFɑ (Grozdanov et al., 2019). Some of these cytokines, like TNFɑ and interleukin-1ɑ, promote reactive astrocytosis, a process which promotes genetic changes in astrocytes that induce a role shift from providing trophic support to neurotoxic activity (Liddelow et al., 2017). This leads to reduced survival of human dopaminergic neurons co-cultured with transformed astrocytes. The presence of these toxic astrocytes were also observed in the post-mortem tissue of PD patients, including in the substantia nigra, (Liddelow et al., 2017) suggesting that neurotoxic astrocytes activated by microglial cytokines contribute to dopaminergic cell death in PD.

While inflammation can mediate neurotoxicity and cell death, neuronal damage would further drive inflammation by releasing chemokines and markers that activate microglia and astrocytes. This creates a feed-forward loop that exacerbates inflammation and drives further neurodegeneration (Wu et al., 2005). Neurodegeneration could further compromise BBB through elevated levels of vascular endothelial growth factor (VEGF), which is responsible for angiogenesis during states of neuroinflammation. VEGF is a potent disruptor of BBB function and has been found to be elevated in the substantia nigra of PD patients (Rite et al., 2007; Wada et al., 2006). BBB damage could further allow systemic inflammation to infiltrate the brain and exacerbate neuroinflammation. This cycle would drive neurodegeneration in susceptible brain areas in PD, such as the substantia nigra (Figure 1). A higher presence of microglia in the substantia nigra compared to other regions in the brain could explain the increased susceptibility of dopaminergic cells in this region to microglial-mediated neurotoxicity (Qin et al., 2007). Dopaminergic neurons are also inherently more vulnerable to reactive oxygen species due to their low antioxidant capacity. Therefore, they are more readily damaged by the oxidative stress produced by neuroinflammation (Block and Hong, 2005).

Aging remains one of the strongest risk factors in developing PD which may be linked to elevated inflammatory pathways. It has been suggested that microglia become “primed” with advanced age, producing more pronounced inflammatory responses (Perry et al., 2007). As such, aged rodents and non-human primates show increased microglial expression of major histocompatibility complex II (MHCII), a marker of microglial activation (Perry et al., 1993; Sheffield and Berman, 1998). Inducing inflammation by peripheral injection of LPS in aged mice also resulted in more long-lasting pro-inflammatory cytokines and oxidative stress compared to younger adult mice, indicating an exaggerated inflammatory response (Godbout et al., 2005). Cellular senescence, an aging process that promotes cell cycle and genetic changes which alter cell function, is another aging phenomenon contributing to inflammation. Aging cells often exhibit mitochondrial dysfunction, elevated reactive oxygen species levels, and senescence-associated secretory phenotype (SASP) that make them more susceptible to PD-related pathology. SASP includes the release of cytokines and chemokines that activate an inflammatory response (Russo and Riessland, 2022). In addition, the senescence of endothelial cells in the vasculature has been shown to promote BBB dysfunction through reduced tight junction coverage in an accelerated aging mouse model (Yamazaki et al., 2016). Therefore, ageing could contribute to the development of PD by exacerbating inflammation, reducing anti-oxidative capacity, and disrupting BBB function. Therefore, systemic inflammation that originates from the gut or other sources may disrupt the BBB integrity, allow the entry of pro-inflammatory cytokines, and induce neuroinflammation. Inflammation also intersects with the aggregation of *α*-Syn, the hallmark protein implicated in PD.

## 4 α-Synuclein aggregation and transmission

**Figure 2:**
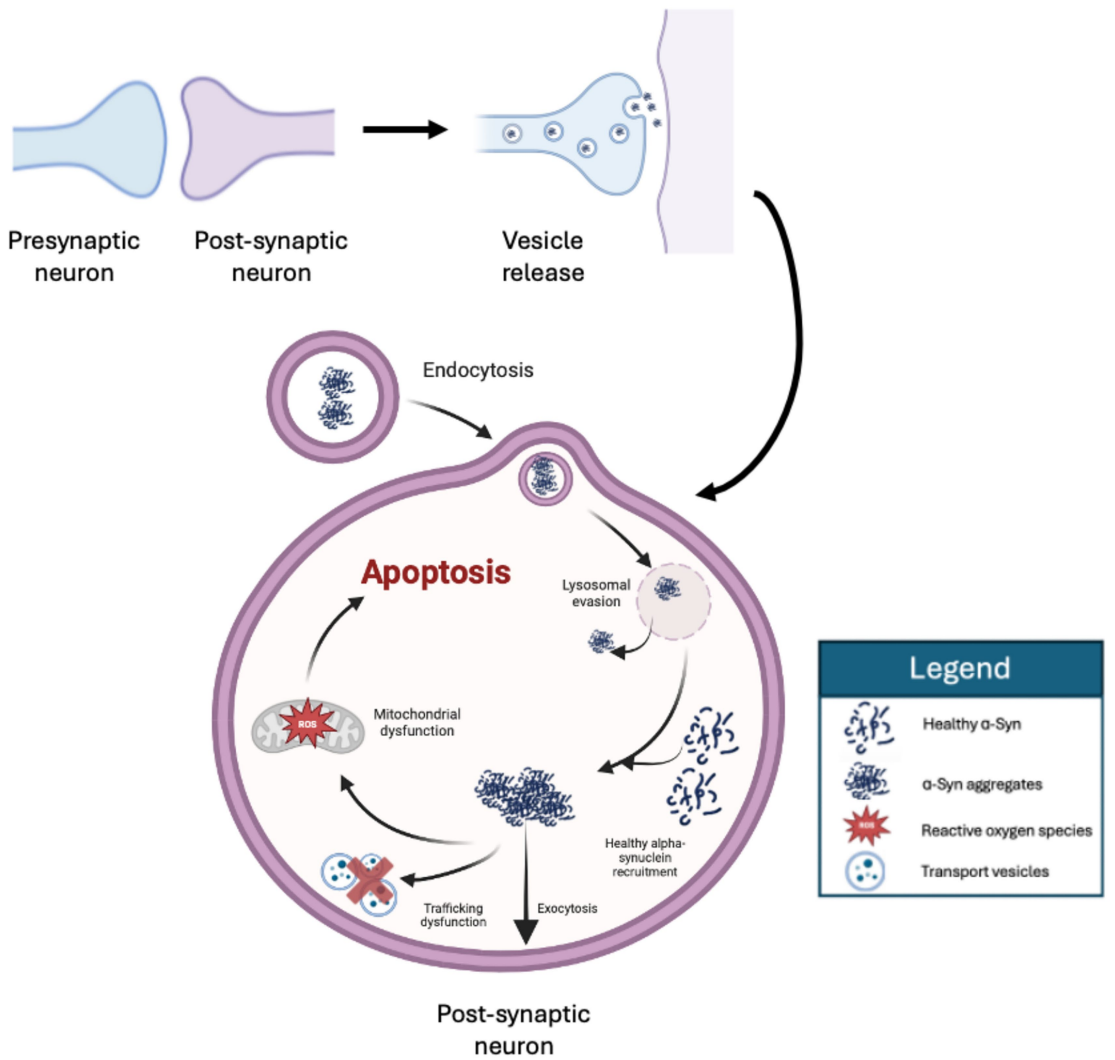
The donor-recipient transfer of aggregated α-Synuclein. Alpha-Synuclein aggregates can be transferred from infected donor cells to neurons in close vicinity, particularly those with synaptic connections. α-Synuclein aggregates leave the donor neuron through different mechanisms, like exocytosis, and are taken up by the recipient neuron via receptor-mediated endocytosis. Once inside the healthy recipient, the aggregates impair lysosomal function and escape degradation via lysosome rupture. In the cytosol, aggregates recruit the cell’s endogenous monomeric α-Synuclein and cause further aggregation. The accumulation of aggregates disrupts cell homeostasis, impairing Golgi-mediated trafficking, and mitochondrial function, leading to reactive oxygen species production and cell apoptosis. Created in BioRender. Fraser, P. (2025) https://BioRender.com/i0zlebg

α-Syn pathology spreads in a transneuronal prion-like fashion, where the aggregates are infectious protein “seeds” that can be transferred between donor and recipient cells (Figure 2). This process leads to the recruitment of endogenous monomeric *α*-Syn into the oligomerized form and amplifies aggregation (Lopes et al., 2022; Rahayel et al., 2022). It has been shown *in vitro* that *α*-Syn localizes to intracellular vesicles and can be secreted, even in aggregated form (Lee et al., 2008). Extracellular α-syn in increased by neuronal activity suggesting that α-syn release is regulated, at least in part, some form of physiological secretion (Yamada and Iwatsubo, 2018). Aggregates could be released through unconventional mechanisms that are independent of the Golgi and the endoplasmic reticulum like exosome-mediated vesicular exocytosis and tunneling nanotubule-mediated transfer through intercellular channels (Emmanouilidou et al., 2010; Abounit et al., 2016; Danzer et al., 2012). Exogenous *α*-Syn can also be internalized by neighboring cells through mechanisms like phagocytosis or receptor-mediated endocytosis (Choi et al., 2021; Lee et al., 2008; Sung et al., 2001). Once in the cell, aggregates enter the endocytic pathway and are found in endosomes and lysosomes to be degraded (Karpowicz et al., 2017; Konno et al., 2012). Investigations have also demonstrated that *α*-Syn aggregates in primary neuron cultures result in vesicular rupture in the endolysosomal pathway, coinciding with an increase in reactive oxygen species (Freeman et al., 2013; Chen et al., 2013; Samuel et al., 2016). Similar results were shown in other investigations which demonstrated that lysosomal rupture by *α*-Syn aggregates was subsequently followed by the aggregation of endogenous α-Syn (Kakuda et al., 2024). The exact mechanisms of lysosomal vesicle rupture remain unknown, however, studies have suggested aggregate-mediated membrane permeabilization, curvature induction, and oxidative stress-mediated membrane damage as possible causes (Senol et al., 2021; Varkey et al., 2010; Volles and Lansbury, 2002). Seeding aggregation disrupts normal neuronal physiology through impairing proteasome function which maintains proteomic homeostasis (Popova et al., 2021; Zondler et al., 2017), disturbing cellular trafficking mechanisms (Cooper et al., 2006; Wang and Hay, 2015), and impairing mitochondrial function (Geibl et al., 2023; Lurette et al., 2023; Ramezani et al., 2023), resulting in oxidative stress (Figure 2).

α-Syn aggregates clearly play a critical role in the pathogenesis of PD and oligomers have been proposed to be more toxic than mature fibrillar aggregates (Mori et al., 2006; Sidhu et al., 2004; Tarutani et al., 2016). Additionally, *α*-Syn strains that create stable pS129 deposits are less toxic than those that are more readily destabilized (Lau et al., 2020). Furthermore, it has been found that α-Syn pathology precedes inflammation and glial activation (Izco et al., 2021). These data cumulatively suggest that α-Syn aggregation, mitochondrial dysfunction, and ROS generation all converge and cooperate toward neuronal death. As such, α-Syn aggregates and oligomers correlate with neuronal death and symptoms based on the affected areas (Kövari et al., 2003; Wakabayashi et al., 2006). This allows the aggregates to serve as a detectable and quantifiable measure of disease progression.

**Figure 3:**
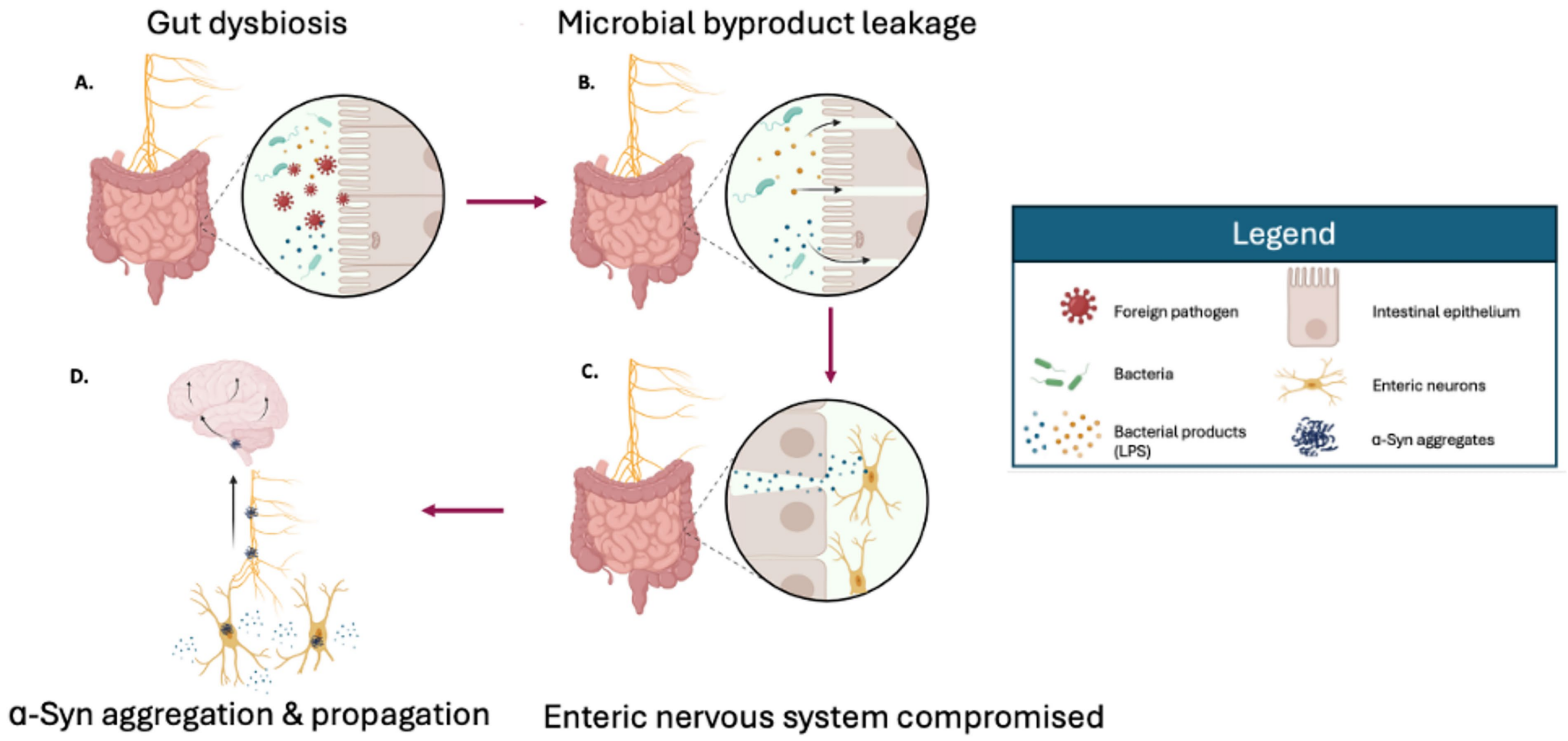
An overview of the gut etiology of Parkinson’s disease. A foreign pathogen **(A)** causes the intestine wall to become permeable **(B)**, allowing microbial products and other intestine contents to cross over the intestine wall and interact with neurons that are situated in the underlying tissue **(C)**. These microbial products interfere with the normal function of neurons and cause the formation of α-Synuclein aggregates **(D)**. These aggregates can move from the digestive system to the brain via the vagus nerve, which connects the brain to internal organs. Created in BioRender. Fraser, P. (2025) https://BioRender.com/jqu4fxg

Neuron-to-neuron transfer of aggregates provide a mechanism for pathology to migrate from the gut to the brain via the vagus nerve (Figure 3). Indeed, *α*-Syn aggregates have been observed in the vagus nerve in pre-clinical studies. Mice injected in the intestine with PD brain lysates containing *α*-Syn aggregates showed a time-dependent transport along the vagus nerve, ultimately reaching the brainstem (Holmqvist et al., 2014). Subsequent investigation demonstrated that injecting pre-formed *α*-Syn fibrils into the intestine of mice resulted in neurotoxicity in the brain and motor deficits (Challis et al., 2020; Van Den Berge et al., 2021); importantly, these studies showed *α*-Syn pathology did not progress beyond the brainstem unless the animals were aged. The age-dependent gut-brain propagation can be circumvented by α-Syn overexpression (Van Den Berge et al., 2019). The gut-to-brain spread of pathology and the resulting motor and cognitive deficits were prevented by truncal (full) vagotomy and in α-Syn deficient mice, highlighting the importance of the vagus nerve for the gut-brain transfer of pathology and the necessary recruitment of endogenous α-Syn substrate for this process (Kim et al., 2019). Indeed, in humans, α-Syn aggregates were observed in post-mortem cervical vagus nerve samples of PD patients (Mu et al., 2013). Additionally, two large cohort studies of vagotomized humans also showed a lower incidence of PD development for individuals that underwent truncal, but not selective (partial) vagotomy at least 5 years prior to diagnosis (Liu et al., 2017; Svensson et al., 2015). Together, the prion-like spread of *α*-Syn along the vagal pathway is a key event in the propagation of pathology to the brain.

It is important to note that the gut-first model does not apply to all PD cases since some patients report GI symptoms without protein aggregation in the brain and others have protein aggregation without clinical GI symptoms (Gaig et al., 2007; Parkkinen et al., 2005). Clinically, not all patients experience premotor GI symptoms (Yu et al., 2018). The modern body-first and brain-first models of PD imply two subtypes of PD, each with unique patterns of *α*-Syn spread that ultimately reach and cause neuronal death in the SN (Borghammer and Van Den Berge, 2019; Borghammer, 2021). This is substantiated by the conformer strain hypothesis which states that different conformations of misfolded α-Syn lead to strains with unique aggregation kinetics, resistance to degradation, and patterns of spread in the brain. For example, aggregates generated from α-Syn monomers in low and high salt concentrations showed different resistance to proteinase K, depolymerization thresholds, and toxicity levels *in vitro* and *in vivo* (Lau et al., 2020; So et al., 2024; Peelaerts et al., 2015). The spread of pathology and motor disruptions in the animals inoculated with α-Syn aggregates also occurred in a strain-dependent manner wherein fibrils formed in high salt conditions caused greater detriment, leading to shorter lifespans (Lau et al., 2020; So et al., 2024). As such, it is possible that the body-first and brain-first subtypes of PD, having different sites of origins, are characterized by different strains of PD. Nevertheless, the GI tract is affected in about 50–70% of PD patients and remains a therapeutic target, even among patients of brain-first PD as GI dysfunction occurs in their later stages of disease (Gan et al., 2018; Borghammer and Van Den Berge, 2019).

Therefore, propagation patterns in the brain can be diverse, as expected with the heterogenous nature of PD, and can be influenced by factors like α-Syn strain, cell vulnerability, and anatomic connectivity. These findings highlight the need for PD therapeutics that can address the heterogenous nature of PD pathology and symptoms as well as target not only the brain but also peripheral organs. The difficulty of simultaneously targeting the central and peripheral neurons can be addressed by adeno-associated virus-mediated gene delivery.

## 5 Gene transfer to the nervous system by systemic AAV

As discussed in the previous sections, the early appearance in the periphery of α-Syn aggregation and inflammation in some PD cases suggests potential benefits of initiating therapeutics to target peripheral sites. Multiple anti-inflammatory drugs have been tested and shown to be beneficial in different animal models of PD. However, although benefits on motor symptoms and inflammation were observed, these are not permanent and the spread of brain pathology is not completely halted (Çınar et al., 2022). To halt the feed-forward loop between neuroinflammation and α-Syn aggregation, synucleinopathy must also be addressed. Delivering gene therapies that can reduce α-Syn pathology in neurons has also shown positive improvements in motor symptoms, cognition and clearing α-Syn aggregates. Among the beneficial gene products are nucleotides that reduce the amounts of SNCA mRNA like microRNAs or short hairpin-RNAs, as well as proteins that drive the clearance of native or fibrillar α-Syn like intrabodies (Menon et al., 2021). The mechanisms of action of these therapies are beyond the scope of this review. This review focuses rather on the delivery of gene therapy vectors to the gut and the brain, specifically by adeno-associated viruses (AAV).

AAV has been an attractive option to use for gene delivery for numerous reasons. First, AAV has innately low immunogenicity as it cannot replicate without adenovirus. Second, it enables efficient gene delivery of sequences up to approximately 4.5 kbp (Hermonat and Muzyczka, 1984). Importantly, in the case of PD, the delivered transgenes have long-term expression even in post-mitotic cells (Royo et al., 2008). This allows a single transduction event to be beneficial throughout the duration of the neuron’s lifespan. Lastly, AAVs delivered in the bloodstream can transduce PD-related organs. Together, this paints a possible future where multiple PD-related organs are treated with a single intravenous injection of AAV-anti-*α*-Syn gene therapy.

Many studies that test gene transfer to the brain usually use direct injection of the gene carried by the AAV1 or AAV2 serotype (Haery et al., 2019). In developing an AAV-mediated gene therapy for PD, preclinical tests have attempted AAV brain transduction through the bloodstream. Among all the naturally occurring AAV serotypes, AAV9 has the best ability to transduce the BBB from the circulation. However, AAV9 may only pass through the BBB with high doses (Gray et al., 2011), in neonates whose BBBs are still underdeveloped (Foust et al., 2009), or with the help of magnetic resonance imaging-guided focused ultrasound (FUS) with microbubbles which can transiently increase the permeability of the BBB (Thévenot et al., 2012). Indeed, mice that received an intravenous dose of AAV9-SNCA-shRNA and FUS showed transgene expression and reduced α-Syn in the FUS-targeted brain areas (Xhima et al., 2018).

To improve AAV9 BBB penetration, a library of AAV9 derivatives was engineered using the Cre-recombinase-dependent AAV targeted evolution (CREATE) method to evolve AAV9 selective for improved brain transduction following intravenous administration (Deverman et al., 2016). Briefly, the CREATE method involves making a library of AAV9 derivatives with evolved capsids. The edited AAV9 library is then tagged with a loxP sequence and injected into transgenic mice that express Cre-recombinase in predefined cell types. PCR is then performed to determine which AAV9 variant transduced the Cre-positive cells. By selecting for transduction in Cre-positive neurons, selection is biased toward those that bypass the BBB.

One of the first BBB-penetrant AAV9 derivatives is AAV-PHP. B (hereafter just PHP. B), known for its brain transduction capabilities (Deverman et al., 2016). An intravenous dose of PHP. B-GFP transduces the entire brain and spinal cord significantly more effectively than the parent AAV9, even at a tenth of AAV9’s dosage. Additionally, PHP. B exhibited significantly less off-target peripheral expression in the pancreas and adrenal gland, with a decreasing trend observed in the liver (Deverman et al., 2016). Further evolution of PHP. B by CREATE led to the development of AAV-PHP.eB (enhanced/evolved brain transduction). PHP.eB demonstrates even greater BBB penetrance and neuronal transduction while maintaining similar astrocyte tropism compared to its PHP. B predecessor (Chan et al., 2017). However, because PHP. B and PHP.eB can transduce the entire brain, they lack spatial resolution which is a critical consideration when designing treatments. If greater localization of gene therapy is required, AAV9 with FUS could be utilized.

Gene delivery to the BBB is also important to rescue its integrity in PD patients who have increased BBB permeability (Al-Bachari et al., 2020). Among the first PHP variants that were discovered is PHP. V1 which showed greater endothelial cell transduction than AAV9 (Ravindra Kumar et al., 2020). Building on this, an improved multiplexed-CREATE (M-CREATE) method was used on AAV9 to create AAV-X1 and -X1.1 which display enhanced selectivity for BBB endothelial cells and avoidance of liver transduction (Chen et al., 2023). Additionally, these two AAV vectors allow for multiple AAV gene delivery and serotype switching from the blood to the BBB which avoid triggering immune responses from the subject. A potential therapeutic strategy for PD was tested by the creators of AAV-X1 and -X1.1. The endothelial cells were turned into a “biofactory” by the transduction of AAV-X1 carrying genes for the matricellular protein Hevin which is helpful for neuronal and astrocytic health (Chen et al., 2023). This biofactory strategy can be used to create BBB resistance against systemic inflammation in PD or other neurodegenerative diseases that also damage the BBB.

In the context of the body-first model of PD, gene delivery to the enteric neurons must also be considered. Once again, AAV9 displays the highest transduction levels in enteric neurons and glia over the other naturally occurring AAVs (Gombash et al., 2014). The development of AAV9 derivative, AAV-PHP. S, which, although not highly efficient at crossing the BBB, exhibits robust expression in the spinal cord and sensory afferents leading to the brainstem (Chan et al., 2017). PHP. S demonstrates enhanced neuronal transduction compared to AAV9 in the dorsal root ganglia (DRG) of the spinal cord, cardiac ganglia, and both the myenteric and submucosal plexuses of the ENS, all relevant to the body-first subtype of PD. In the ENS, PHP. S shows superior transduction efficiency and uniformity along the length of the small intestine compared to AAV9 (Chan et al., 2017). Systemic injection (iv) of PHP. S-GBA1 transduced enteric neurons and increased duodenal GCase expression (Challis et al., 2020). This treatment mitigated the pathology induced by *α*-syn pre-formed fibrils and restored gut function to healthy wild-type levels.

Since PD leads to dysfunction of both the CNS and ENS, optimizing gene delivery to both the brain and gut could be beneficial. This can be done through MaCPNS1 and MaCPNS2, two derivatives of PHP. S that target the brain and small intestine with reduced liver expression (Chen et al., 2022). Additionally, both vectors exhibit approximately a two-fold increase in tropism for the dorsal root ganglia of the spine and nodose ganglion of the vagus nerve. The ability to target multiple peripheral nerves without significant liver tropism is beneficial as AAV’s natural high affinity for the liver reduces the bioavailability for the target cells and may risk liver damage (Flotte, 2021; Zincarelli et al., 2008). Lower liver tropism may also decrease the required AAV dose and help avoid undesired immune responses that could exacerbate inflammation (Mingozzi and High, 2013).

Together, the current understanding and engineering of AAVs sets the scene for a gene delivery therapeutic for PD that could be administered by a single or few intravenous injections. The treatment serotype used could also be personalized to the patient based on the location of the disease (Table 1). For example, PHP. S-like serotypes may be used for patients in the early stage of disease that follows the gut-to-brain model while those who follow a brain-centric disease progression may benefit more from PHP.eB-like serotypes or the combination AAV9 and FUS. MaCPNS-like serotypes may be beneficial for patients in whom central and peripheral symptoms are observed. If the BBB is the target of gene delivery, then serotypes with the properties of AAV-X1 and -X1.1 may be used. Importantly, all the possible options mentioned avoid highly invasive brain or intestinal injections.

**Table 1:** Summary table of AAV serotypes with tropism to PD-related organs via the bloodstream.

AAV serotype	Organ tropism after intravenous delivery	Source
AAV9	Brain (with the aid of low-intensity focused ultrasound), ENS	Thévenot et al. (2012) and Gombash et al. (2014)
PHP. B and PHP.eB	Brain	Deverman et al. (2016) and Chan et al. (2017)
PHP. V1	Blood brain barrier	Ravindra Kumar et al. (2020)
AAV-X1 and -X1.1	Blood brain barrier	Chen et al. (2023)
PHP. S	ENS, heart, spinal cord	Chan et al. (2017)
MaCPNS1 and 2	CNS, ENS, heart	Chen et al. (2022)

## 6 Conclusion

In many cases, Parkinson’s disease is associated with the presence of α-Syn pathology and pre-motor gastrointestinal symptoms, the neurotoxic effects of pesticides, herbicides, and toxins injected in the gastrointestinal system, and post-mortem patterns of aggregates in brain structures. The observation of pro-inflammatory shifts in the gut microbiome makeup of PD patients suggests a potential source for neurotoxin production and initiation of systemic and neuroinflammatory responses that may drive α-Syn aggregation. Disruptions to the homeostatic state of neurons, their inherent physical connectivity, and chronic inflammatory responses collectively drive neurodegeneration in susceptible dopaminergic cells, primarily through mitochondrial dysfunction. However, key questions remain, including the mechanisms through which dysbiosis and toxins contribute to α-Syn aggregation, whether α-Syn aggregation acts as a defensive mechanism or a pathology driver, how α-Syn bypasses endolysosomal degradation, and the reasons behind the vulnerability of dopaminergic cells. Also, further studies on pre-clinical stage PD patients are required to help elucidate early changes in the microbiota and the extent of the subsequent damage in the enteric nervous system.

Since the body-first model of PD with early peripheral nervous system involvement holds true for a subset of PD patients, a brain-first manner of propagation has also been hypothesized. This is consistent with clinical manifestation where some patients do not exhibit autonomic symptoms prior to motor deficits caused by dopaminergic loss in the striatum and substantia nigra. Deciphering whether different α-Syn strains are involved with the two subtypes could help elucidate the etiology of PD. α-Syn-related pathology is implicated not only in PD, but also in other neurodegenerative diseases, such as dementia with Lewy bodies, multiple system atrophy, and pure autonomic failure (Calabresi et al., 2023). These synucleinopathies have different aggregation types but also overlapping clinical and pathological features, meriting further research into the applicability of the gut-first hypothesis, or parts of it, to other disorders.

The implication of multiple sites of inflammation and α-Syn pathology posits the need of delivering beneficial gene products to the affected sites. Multiple gene therapy strategies that target aggregated α-Syn can reduce inflammation in the targeted sites. However, more research on therapies that avoid invasive brain injections or surgeries is required. To this end, current advances in AAV gene delivery may simultaneously deliver these gene products to multiple PD-related organs, like the gut and brain, through a single intravenous injection. The AAV toolkit discussed in this review also points to the possibility of personalizing the gene delivery treatment, thus addressing the multi-faceted characteristic of PD or other synucleinopathies. Future preclinical and clinical experiments may test whether and how systemic gene therapy may rescue, reduce, delay, or prevent synucleinopathies.
